# The South-to-North Water Diversion Project: effect of the water diversion pattern on transmission of *Oncomelania hupensis*, the intermediate host of *Schistosoma japonicum *in China

**DOI:** 10.1186/1756-3305-5-52

**Published:** 2012-03-20

**Authors:** You-Sheng Liang, Wei Wang, Hong-Jun Li, Xue-Hui Shen, Yong-Liang Xu, Jian-Rong Dai

**Affiliations:** 1 Jiangsu Institute of Parasitic Diseases, 117 Yangxiang, Meiyuan, Wuxi 214064, People's Republic of China; 2 Key Laboratory on Technology for Parasitic Disease Prevention and Control, Ministry of Health, 117 Yangxiang, Meiyuan, Wuxi 214064, Jiangsu Province, People's Republic of China; 3 Dantu District Center for Disease Control and Prevention, 171 Guyang Avenue, Danyang, Zhenjiang City 212028, Jiangsu Province, People's Republic of China

**Keywords:** *Schistosoma japonicum*, Schistosomiasis japonica, The South-to-North Water Diversion Project, *Oncomelania hupensis*, Transmission, Spread, Diversion pattern, China

## Abstract

**Background:**

The South-to-North Water Diversion Project (SNWDP) is the largest national water conservancy project in China. However, the Eastern Route Project (ERP) of SNWDP will refer to the habitats of *Oncomelania hupensis*, the intermediate host of *Schistosoma japonicum*. The present study was aimed at investigating the effects of some factors relating to the water diversion pattern on the spread north of *O. hupensi*s and transmission of *S. japonicum*.

**Methods:**

Marked snails were attached to the floating debris, and then placed on the water surface, the passage of snails through water pumps was observed. Some marked living adult snails were placed under water in the 5 spots, 15, 30, 60, 90 and 120 days later, their survival and transfer under water were investigated. 2, 4, 8, 16, 32, 64 and 128 juvenile snails, with a male: female ratio of about 1, were caged, 1 year later, their reproductions were calculated.

**Results:**

The snails attached on the floating debris at 100-, 50- and 20-cm-distance from the inlet pipe of the big pump (with a diameter of 80 cm), could be absorbed into the pumps, with passing rates of 2.45%, 3.93% and 43.46%, respectively, compared with 72.07% and 91.00% for the snails at 20 cm and 10 cm-distance from the inlet pipe of the small pump (with a diameter of 20 cm). A total of 36,600 marked living snails were put into 5 ponds and ditches, with the water depths of 1-1.6 m, 15-120 days later, no marked ones were found along the ponds and ditches or in the straw packages. The juvenile snails did not reproduce until their density reached up to 8 snails (ratio of male: female of 1)/0.16 m^2^.

**Conclusions:**

During the construction of ERP of SNWDP, the risk of northward spread of schistosomiasis japonica will be decreased or eliminated as long as long-term reliable interventions for snail control are implemented.

## Background

Schistosomiasis is a snail-borne parasitic disease, which affects more than 207 million people in tropical and subtropical regions [1]. In China, schistosomiasis japonica remains endemic in lake regions of five provinces along the lower and middle reaches of the Yangtze River, and in some mountainous regions of Yunnan and Sichuan provinces. Currently, about 0.7 million people living in China are thought to have this disease [2,3]. *Oncomelania hupensis*, the amphibious intermediate host of *Schistosoma japonicum*, is only found in the Chinese mainland at latitudes below 33°15 N [4]. As the geographical distribution of *O. hupensis *defines the areas in China where *S. japonicum *is endemic, control of this snail is currently one of the main approaches used in attempts to interrupt the transmission of the disease [5-10].

The South-to-North Water Diversion Project (SNWDP), the key, national water-conservancy project currently under construction in China, has been designed to optimize the use of the limited water resources in China, and relieve water shortages in the north of the country [11]. The start of one of the main water intakes for the project, that of the Eastern Route Project (ERP), is located in Jiangdu County of Jiangsu Province, in an area heavily infested with *O. hupensis *[12]. The route of ERP will cross Baoying County (at 33°15 N), the current northern limit of *O. hupensis *in the Chinese mainland [13], and then pass northward into non-endemic areas where the snail is now unknown. Following the completion of the project, whether the *O. hupensis *will be carried northward with the water flow, and lead to re-emergence of *O. hupensis *along the route of ERP and their new, more northern habitats and of the resultant expansion of *S. japonicum *endemicity to areas of China north of 33°15 N, is the subject of much research [11,14-18].

**Figure 1:**
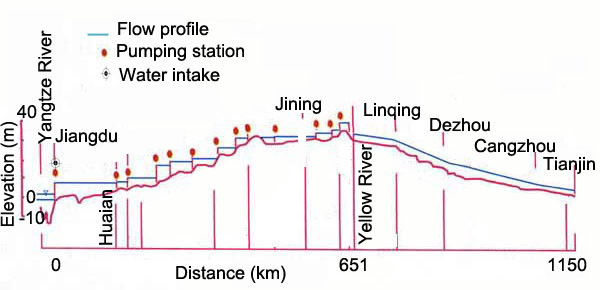
**The diversion route of the Eastern Route Scheme of the South-to-North Water Diversion Project**.

*O. hupensis*, the only endemic intermediate host of *S. japonicum *in China, is both stenothermal and amphibious. Although the adult *Oncomelania *snails are able to live either on wet soil in the riverbank or in water, they are also mostly spread via water, usually on floating debris. While juvenile snails are entirely aquatic and are spread via water from the upper to lower reaches [13]. Wang and colleagues [11] set up caged populations of *O. hupensis *in Xuzhou (34°23 N) and Jining (35°23 N), two areas north of 33°15 N, along the route of the ERP, and found that the snail populations could survive and reproduce in these areas, and retained their infectivity to *S. japonicum*. However, unlike the natural water flow, the ERP makes use of multi-stage pumping stations for diverting water from the south with low elevation to the north of the country with high elevation [12,19] (Figure 1). The aim of the present study was to investigate, during the multi-stage pumping water, the passage of snails through the pumps, the survival of the unattached snails and the reproduction of snails at different densities, so as to provide evidence for assessment of safe operation of SNWDP and implementation of scientific prevention and control interventions.

## Methods

### Study areas

For the experiment of snail movement under water, the study focused on 5 areas. Spot 1 was a fish pond in Gaozi Town, Dantu District of Zhenjiang City, measuring 145 m × 60 m (water depth of 1.6 m), without weeds or aquatic plants. Spot 2 was a natural pond with an area of 120 m × 51 m (water depth of 1.5 m) in Gaozi Town, Dantu District of Zhenjiang City, and no aquatic plants were found. Spot 3 was a rectangular pond (size of 50 m × 70 m, water depth of 1 m) in Shiye Town, Dantu District of Zhenjiang City, without aquatic plants. Spot 4 was a ditch with an area of 200 m × 2 m and a depth of 1.2 m in Shiye Town, Dantu District of Zhenjiang City, no aquatic plants were present. Spot 5 was an irregular pond with a depth of 1.2 m in Shiye Town, Dantu District of Zhenjiang City, and no aquatic plants were found. All of the 5 areas are currently endemic for *S. japonicum*, where *O. hupensis *are distributed in surrounding ponds or ditches.

For the experiment of snail reproduction, a pond with an area of 825.8 m^2 ^was selected in Xinmin Marshland, Gaoyou City of Jiangsu Province (32°42.8 N, 119°23.6 N), which was, historically, an endemic area, and currently is still a snail habitat with low density.

### Snails

*O. hupensis *snails were collected from the rural marshland of Dantu District, Zhenjiang City by picking them up with forceps. Snails were transferred to the laboratory and raised for 2 weeks at a temperature of 25°C. Each snail was tested twice for natural infection using the cercarial shedding method [20]. Since none of the snails had natural infections, active adult snails with 6 ~ 7 whorls were used. Then each snail was marked in red or green color on the apex using a TOYO marker for the subsequent experiments. A snail with a length of < 5 mm was defined as juvenile, each juvenile snail was separated into male and female by microscopical examination, and then was used for the subsequent experiments.

### Snail cages

Cages containing wild-caught, juvenile *O. hupensis *were set up at the study site for investigation of the effect of the snail density on reproduction of *O. hupensis*. Each cage was a cube measuring 40 cm × 40 cm × 40 cm, made of a strong frame of iron wire covered in wire netting (with a mesh size of 1.18 mm). A piece of nylon gauze (with a mesh size of 0.12 mm) was placed across the bottom of each cage to support a 10-cm-thick layer of unpolluted local soil.

### Pumps

Two types of pumps were used in the present study. The bigger one was 80 cm in diameter and 585 r/min in rotational speed, with a power of 80 kW. Another was 20 cm in diameter and 7.5 kW in power.

### Tucknets

Two coniform tucknets were made for collecting the snails that passed through the pumps. The bottom of the both tucknets was made of a strong frame of circular iron wire (with diameters of 85 and 22 cm), which were covered with nylon gauze with a mesh size of 0.12 mm.

### Straw packages

Some straw packages, each weighing 0.5 kg, were made to attract snails.

### Snails passing through the pumps

A total of 30,000 marked snails were attached to the floating debris, 6,000 in each position, and then placed on the water surface at 100- 50- and 20-cm-distance from the inlet pipe of the big pump, and 20- and 10-cm-distance from the inlet pipe of the small pump. Following switching on the pumps, the attached snails would be absorbed into pumps. The tucknets were fixed onto the outlet pipe of the pumps to collect snails.

### Snail movement under water

From 24th, June to 13th, October 2007, a total of 8400 marked snails were placed at the site, 10 m from the north bank and 20 m from the east bank in Spot 1, 6,200 marked snails were placed at the site 2 m from the east bank and 5 m from the north bank in Spot 2, 5,000 marked snails were placed at the site 3 m from the north bank and 5 m from the west bank in Spot 3, 5,000 marked snails were placed at the site 1 m from the bank in Spot 4, 12,000 marked snails were placed at the site 5 m and 6 m from the west bank in Spot 5, each containing 6,000. Straw packages were placed on the water surface of all spots at a distance interval of 10 m. 15, 30, 60, 90 and 120 days later, the marked snails were collected along the pond and ditch bank. The straw packages floating on the water surface were also collected to check the presence of the marked snails.

### Effect of snail density on reproduction

2, 4, 8, 16, 32, 64 and 128 juvenile snails, with a male: female ratio of about 1, were put into snail cages and then sealed with iron. Each cage was placed beside the pond, with the base at an angle of 30° from horizontal, so that a third of the soil layer in the cage was immersed in water. One year later, all cages were removed from the study site, and the snails in the cages were recovered, by sifting the soil in the cages through an 830-m-mesh brass sieve. Those suspected of being dead were tested by observing their responses to being tapped. Reproduction of the snails was recorded.

### Ethical approval

This study was approved by the Ethics Review Committee of Jiangsu Institute of Parasitic Diseases, China (Permission number: JIPDERC2007008).

## Results

### Snails passing through the pumps

The snails, attached to the floating debris, at 100, 50 and 20 cm from the inlet pipe of the big pump, could be absorbed into the pumps, with passing rates of 2.45% (147/6000), 3.93% (236/6000) and 43.46% (2608/6000), respectively. The attached snails at 20 and 10 cm from the inlet pipe of the small pump could also be absorbed, with passing rates of 72.07% (4324/6000) and 91.00% (5460/6000), respectively. It is indicated that a higher possibility of snails passing through the pumps is observed as the initial snail placement distance from the inlet pipe decreases.

### Snail transfer under water

A total of 36,600 marked living snails were put in the 5 study areas, 15, 30, 60, 90 and 120 days later, no marked snails were found along the pond and/or ditch bank. There were also no snails found in the straw packages. However, on February of the following year, there were many dead marked snails found in the mud near the snail throwing sites of spots 4 and 5.

### Reproduction of snails with different densities

2, 4, 8, 16, 32, 64 and 128 juvenile snails were put into snail cages with a male: female ratio of about 1, one year later, snails did not reproduce until densities reached up to 8 snails/0.16 m^2 ^(Table 1).

**Table 1:** Reproduction of juvenile snails with different densities of breeding for 1 year in the field

Juvenile snails (male: female ratio of 1) with different densities (snails/0.16 m^2^)	Group I			Group II		
	
	No. snails collected	No. living snails	Reproduction (%)	No. snails collected	No. living snails	Reproduction (%)
2	2	1	-50	1	1	-50

4	3	3	-25	3	1	-75

8	30	21	162.5	35	27	237.5

16	49	43	168.8	51	47	193.8

32	58	51	59.4	63	56	75

64	118	108	68.8	121	110	71.9

128	229	197	53.9	243	201	57

## Discussion

Schistosomiasis is a snail-transmitted, water-borne devastating neglected tropical disease [21,22]. In China, schistosomiasis japonica is endemic in 7 provinces with about 0.7 million people infected with the causative agent, *S. japonicum *[2]. Historically, the geographic distribution of the disease was restricted south to the 33°15'N latitude, governed by the distribution of its intermediate host snail, i.e. *O. hupensis *[4,13]. Unlike *Biomphalaria *and *Bulinus *snails that are entirely aquatic, *O. hupensis *is both stenothermal and amphibious. There were several studies reporting the transmission intensification or introduction of schistosomiasis into non-endemic areas following water resource development [23-27]. Considering schistosomiasis is widely distributed and the infection rates can change promptly, it is considered as a sensitive indicator disease for monitoring ecological transformation [28]. Steinmann and colleagues [1] conducted a systematic literature review and meta-analysis in 58 studies which attempt to examine the relation between water resource development projects and schistosomiasis, and it is concluded that the development and management of water resources is an important risk factor for schistosomiasis.

SNWDP is currently the largest water resource development project under construction in China [11]. Not only is the water intake of the ERS located in an *O. hupensis *habitat, but the route of the completed ERS will cross many other historical breeding sites for this species, to the north where the disease is currently not endemic [12,13]. The impact of ERP of on the transmission of *S. japonicum *in China is focused on the effects on distribution of *O. hupensis *[14,29,30], which includes 3 key points, absorption of snails into water-diversion route by the pumps, movement and survival of snails that fall into water, and the reproduction of low-density snails and formation of new populations. The possibility that, by carrying the intermediate host northwards, the SNWDP will lead to a northern expansion of schistosomiasis japonica in China has therefore been investigated [29-36]. Survival and reproduction of snails in areas north of 33°15 N has also been reported [11,37-39], however, the assessment of the impact of the spread of snails remains unclear. The study described here, therefore, was designed to investigate whether the attached snails on the floating debris fell into the river course and then entered into the next stage, the possibility that the unattached snails survived in deep water and then moved to the riverbank, and the reproduction of snails at different densities in field simulated experiments.

Our findings showed that, if the snails were absorbed into the pumps, they would the pass through the pumps. The percentage of snails passing through the pumps was associated with the snails entering into the inlet pipes. While the snails were put into a distant point from the inlet pipe, they were more affected by the rotating eddy current effect induced by the water flow during the movement of floating debris to the inlet pipe, resulting in the snails being more likely to be separated from the floating debris. Therefore, snail control interventions should be implemented around the water intake to reduce the snail density and decrease the possibility of snails entering the water-source area via floating debris. In addition, the hydraulic structures constructed in water intake and on the water-diversion route, such as flood-gates, fences and stilling pools, would cause the sedimentation of many snails, and decrease the risk of snails being absorbed into pumps [12,15,19,40-44].

In the multi-stage pumped water, under the effect of pumps, the adult snails were separated from the floating debris and fell into the deep water while passing through the pumps. Whether the unattached snails can survive in the deep water and transfer to the bank, is an important ecological issue. It has been reported that, dyed snails were put into a streamlet, and 4 days later, they were found along the water line [45]. However, our present findings showed that, a total of 36,600 marked living snails were put into 5 spots and 4 types of water body at different times (from June to October), after a period of 120 days, there were no marked living snails found along the pond or ditch bank or in the straw packages, but plenty of marked dead snails were found in the mud of the dried study areas. Adult *O. hupensis*, an amphibious and freshwater species, hardly ever survives and reproduces under water [13,46,47]. The current study showed that although adult *O. hupensis *can move via foot [13], generally the adults rarely transfer to the bank and survive. It has also been indicated that there were no snails found in the fence of the pumping station and the water-transporting channel in Jiangdu pumping station, the water intake of ERP [40]. Further study should be carried out to investigate the effect of environmental differences between the streamlet and pond on the spread of snails.

Juvenile *O. hupensis *snails are entirely aquatic and are spread via water [13]. There is currently no sufficient evidence to prove the threshold of the snail density for reproduction to new snail habitat. Our present study indicates that snails are likely to be reproduced to form a new population when the density of the juvenile snails reaches 8 snails (ratio of male to female is 1)/0.16 m^2^. However, this is only a preliminary finding, future experiments should be conducted for further verification.

Overall, it is thought that, inevitably, although the attached snails will enter into the river course during the multi-stage pumped water of ERP of SNWDP, under the effect of pumps, the attached snails will be separated from the floating debris, and fall into the deep water, and result in a small possibility of transferring to the bank and survival. In addition, the hydraulic structures constructed on the water-diversion route, such as floodgates, fences and stilling pools, will cause the sedimentation of many snails, again restricting the spread of adult snails via the pumped water [12,40,41]. Juvenile snails are spread via water, and reproduce while reaching a certain density.

## Conclusions

During the construction of ERP of SNWDP, it is very likely to reduce or eliminate the risk of snail spread north if effective snail control interventions are implemented to control snail spread and reduce snail density.

## Competing interests

The authors declare that they have no competing interests.

## Authors' contributions

YSL, WW and JRD conceived and designed the study. YSL and JRD collected baseline data. X.H.S. provided logistical support for part of the fieldwork. YSL, WW, HJL, XHS, YLX and JRD took part in all of the fieldwork. WW carried out the statistical analysis and interpretation of the data and prepared the manuscript. YSL and JRD revised the manuscript. All authors read and approved the final manuscript.
